# Quantifying Sheep Behaviour Using a 3D Accelerometer: A Proof-of-Concept for Objective Stress Assessment

**DOI:** 10.3390/s26041169

**Published:** 2026-02-11

**Authors:** Stephanie Janet Schneidewind, Mohamed Rabih Al Merestani, Sven Schmidt, Wolfgang Waser, Tanja Schmidt, Mechthild Wiegard, Uwe Schmidt, Christa Thoene-Reineke

**Affiliations:** 1 Institute of Animal Welfare, Animal Behaviour and Laboratory Animal Science, School of Veterinary Medicine, Freie Universität Berlin, 14163 Berlin, Germany; stephanie.schneidewind@outlook.de (S.J.S.); tanja.schmidt@bfr-bund.de (T.S.); melad96@web.de (M.W.);; 2 Department of Biosystems Engineering, Albrecht Daniel Thaer Institute of Agricultural and Horticultural Sciences, Faculty of Life Sciences, Humboldt University of Berlin, 10117 Berlin, Germany; 3 Frankenfoerder Research Society mbH, 14943 Luckenwalde, Germany; 4 BITSz Electronics GmbH, 08060 Zwickau, Germany

**Keywords:** sheep behaviour (*Ovis aries*), sensor, triaxial accelerometer, behaviour classification algorithms, stress detection, welfare monitoring, rumination

## Abstract

**Highlights:**

**What are the main findings?**

- A novel, miniaturised three-dimensional accelerometer system incorporating a nRF52832 microcontroller was developed with a primary focus on rumination detection. In addition to this core function, the system enables classification of resting/idling behaviour and shows potential for detecting eating behaviour following further technical refinement.
- The duration of rumination and resting/idling changed significantly when sheep were separated from their familiar group and relocated to an unfamiliar environment in pairs, which was defined as a “potential stressor.”

**What are the implications of the main findings?**

- Deviations in rumination and resting/idling from individual baseline behaviour may be suitable for triggering automated alerts, as such deviations may indicate stress-related responses. However, validation in larger cohorts of sheep is required to confirm this applicability.
- Automatic behaviour detection represents a promising approach for early stress identification and reliable welfare assessment in both laboratory animal and livestock settings.

**Abstract:**

Continuous digital monitoring of sheep behaviour shows potential for early stress detection. In Part 1 of this study, a novel accelerometer-based behaviour-recognition system using a nRF52832 microcontroller with Bluetooth wireless data transfer was developed and validated. A dedicated algorithm was developed to focus on the automatic detection of rumination, which also enables the classification of resting/idling and eating. The system achieved accuracies of 0.87 (rumination), 0.90 (resting/idling), and 0.86 (eating). Specificities were 0.87, 0.95, and 0.94; sensitivities 0.89, 0.80, and 0.60; and precisions 0.79, 0.88, and 0.73, respectively. In Part 2, four sheep were continuously monitored for 24 h to establish baseline behavioural durations. Animals were then relocated in pairs to an unfamiliar enclosure for a further 24 h observation period. Relocation resulted in a significant reduction in rumination time (−45.6%, *p* < 0.05) and a significant increase in resting/idling (+47.9%, *p* < 0.05), while time spent eating decreased but did not reach statistical significance (−36.2%). These findings indicate that detecting deviations from baseline rumination and resting/idling durations may serve as suitable ethological parameters for automated, sensor-based stress alerts. With further technical refinement and validation, the developed system shows strong potential as a reliable, non-invasive tool for monitoring key sheep stress indicators.

## 1. Introduction

Accelerometer-based behaviour monitoring shows strong potential for both laboratory animal science and precision livestock farming. It enables objective measurement of stress and well-being, supporting efforts to minimize avoidable stress and promote improved welfare outcomes [1,2]. In research settings involving laboratory animals, avoidable stress can compromise ethical standards and alter physiological responses, which can potentially confound experimental results and reduce the reproducibility of animal studies [3]. Early, objective detection of stress-induced behavioural changes is therefore of scientific importance. For sheep, as prey animals, subtle behavioural indicators often precede overt signs of pain and distress [4], making reliable behavioural monitoring a viable tool for welfare assessment. However, manual behavioural observation is labour intensive, time consuming, and susceptible to observer bias, and the mere presence of an observer may influence the animals’ behaviour, thereby limiting its value for continuous welfare assessment [5]. Automated, sensor-based systems offer a promising alternative, which enable continuous, non-invasive, and objective monitoring. Three-dimensional (3D) accelerometers, in particular, have emerged as a valuable tool for classifying behaviours such as rumination, resting/idling, eating, standing, and lying down [6].

Part 1 of this study aimed to develop and validate a novel behaviour-recognition system based on a three-dimensional (3D) accelerometer. Commercially available accelerometers designed and marketed for sheep often provide good measures of general motion and activity. They can reliably classify key activities, including grazing, walking, and standing [7]. Previous studies have adapted consumer or livestock sensors (e.g., sensors developed for humans or cattle) to evaluate the reliability of such systems to classify ingestive behaviours [8]. To the authors’ knowledge, there is currently no commercially available halter-mounted sensor system designed specifically for sheep rumination detection. As sensor performance can be influenced by factors such as wool length, head size and shape, individual behaviour, and sensor placement, a species-specific design is essential [8]. Devices originally developed for cattle may be relatively bulky, causing discomfort or behavioural disturbances in sheep [9]. To make them more useful for extended behavioural monitoring, sensor designs for sheep require miniaturisation and energy efficiency [8]. Previous studies have assessed the performance of accelerometer-based systems in detecting a variety of sheep behaviours. The sensitivities of the systems cited here ranged from 77.2 to 87.0%, the accuracies from 76.1 to 96.7%, the specificities from 89.2 to 98.9%, and the precisions from 75.0 to 92.0% [7,10,11,12,13,14,15,16]. A systematic review published in 2023 [8] provides an overview of sensor-based rumination detection in sheep, including descriptions of device types, testing environments, animals used, sensor positions, classification methods, and system performance values. The performance of accelerometers in distinguishing low-activity behaviours (resting/lying/standing) is high, as exemplified by the 96% precision and specificity achieved for resting in the study by Ikuroir et al. [17].

Part 2 of this study assessed the suitability of three behavioural parameters—rumination, resting/idling, and eating—for their potential use in triggering automated sensor-based alerts. Establishing which behaviours reliably reflect changes in the animals’ state was essential before developing a system to generate alerts. The actual design of the alert-triggering system was not part of this study but is intended for future practical applications. Previous studies have examined a variety of behaviours as ethological indicators of sheep welfare. In particular, rumination has been investigated as a marker of pain and stress. Its duration reflects both feeding physiology and stress levels, with reductions reported under a range of moderate to severe stressors. A 2012 study found rumination to be the most ‘sensitive’ behavioural indicator of stress in sheep [18]. The authors examined the effects of immobilisation, surgery, and changes in feeding regimes on feeding, rumination, standing, and resting duration. They validated these behavioural changes using haematological and biochemical measures. They found that when sheep spend more than 40% of their rumination time in the standing position, it reliably indicates reduced well-being. One study found that ewes ruminated for 129 min less per day when placed in social isolation, compared with socially housed control animals. This was accompanied by elevated cortisol levels and increased stress-related behaviours [19]. A 2018 study reported similar reductions in rumination and feeding, alongside increased standing and physiological stress markers, following 24 h of isolation [20]. A study in which sheep were relocated and regrouped with unfamiliar conspecifics reported a significant decrease in rumination in animals that were regrouped with new conspecifics, compared to the control group [21]. A systematic scientific assessment of which ethological parameters are most suitable for triggering automated, sensor-based alerts under stress conditions is still lacking. This study looks into separating sheep from their familiar group and moving them to a new enclosure in pairs, in order to determine the suitability of rumination, resting/idling, and eating behaviours for digital analytics. The ultimate goal is to use baseline behavioural durations as a reference for identifying potential stress through the detection of deviations from this baseline. Comparing the duration and frequency of behaviours under baseline and potentially stressful conditions offers a promising approach for improving animal welfare, as it enables automated alerts when deviations from individual or group baseline patterns are detected [7]. To the authors’ knowledge, this has not yet been examined in the scientific literature for sheep. The aforementioned manipulation is defined as a “potential stressor”, since the level of stress response experienced by sheep is unclear. The procedure is still expected to induce some stress, since any novel husbandry operations, although routinely performed in either context of sheep housing, may result in arousal and frustration in sheep [21]. This procedure does not constitute an experimental intervention in the sense of animal experimentation, but rather a husbandry-related measure, as the authors intentionally avoided inducing substantial stress at this proof-of-concept stage. Accordingly, the authors acknowledge that the level of stress experienced by individual sheep may have varied and that, in some cases, the procedure may not have elicited stress associated with a negative affective state.

The overarching goal of the study is to improve stress assessment in sheep by identifying behavioural parameters that can be quantified, can be reliably detected by an accelerometer-based system, and are responsive to potential stressors. To achieve this, Part 1 focused on developing and validating a 3D accelerometer system with tailored algorithms for rumination detection designed specifically for the morphology of sheep, while Part 2 assessed the suitability of three key behaviours for triggering automatic stress-related alerts.

## 2. Materials and Methods

### 2.1. Animals and Research Site

Ethical approval for the study was obtained from the State Office for Health and Social Affairs (Landesamt für Gesundheit und Soziales, LAGeSo Berlin) under registration number StN 014/20. The authorities did not classify the study as an animal experiment by German law. Five adult, multiparous, non-lactating, non-pregnant, non-fistulated female German Black-Headed Mutton sheep between three and seven years of age were included. The animals had an average body weight of 120 kg (range: 110–130 kg). All the sheep were housed together under the same management conditions. All of the sheep were in excellent health. One animal (No. 3) exhibited atypical jaw movements due to missing teeth. However, it still had a functional rumen and a good body condition score and was under continuous veterinary observation. There was therefore no reason to exclude the animal from the study due to health concerns. In practical applications, both livestock populations and sheep in a laboratory setting are heterogeneous, with some animals presenting dental defects or other anatomical variations that influence chewing dynamics, which makes the inclusion of this animal helpful.

The five sheep used in this study were housed together in a rectangular enclosure at Freie Universität Berlin. Prior to the start of the study, the facility was approved by the local authority for experimental purposes (Landesamt für Gesundheit und Soziales). The enclosure, which measured approximately 28 m^2^ (3.6 m × 7.4 m), was straw-bedded and had ample natural and artificial lighting. When trials were not being conducted, the sheep were granted access to an outdoor enclosure for several hours each day.

Initially, the animals underwent a quarantine period and were acclimatised to their new housing environment. Positive reinforcement training (clicker training) was implemented to familiarise the animals with regular handling, tactile contact, and wearing a halter. Throughout Parts 1 and 2 of the study (September 2021 to April 2023), the composition of the groups remained unchanged, as did feeding and management practices, with the exception of the separation and relocation described in Section 2.4.1. The sheep were fed hay and pellet feed twice daily (at 06:00 and 14:00) with continuous access to fresh water. Other husbandry practices (e.g., cleaning and maintenance of pens or enclosures, regular health checks, and grooming and hoof care) adhered to standard procedures. The same animals were used for both parts of the study: model development and to study the behavioural changes induced by a potential stressor.

### 2.2. Sensor Device and Measuring System

#### 2.2.1. Development of a Mobile Sensor System for Automated Behavioural Monitoring in Sheep

The sensor prototype and measurement system used in this study were specifically developed for this research project in the workshops of the project partner, BITSz Electronics GmbH, in Zwickau, Germany. The mobile component of the system comprised a ADXL345 accelerometer sensor device (Analog Devices, Munich, Germany), a base transducer unit integrated in the microcontroller nRF52832 (Nordic Semiconductor, Trondheim, Norway) with a standard 2.4 GHz antenna, a laptop (Lenovo, Beijing, China) equipped with dedicated software developed by BITSz electronics (Zwickau, Germany), and a halter from Albert Kerbl GmbH (Buchbach, Germany).

Between January and August 2021, the Department of Biosystems Engineering at Humboldt University of Berlin and the Institute for Animal Welfare, Animal Behaviour and Laboratory Animal Science at Freie Universität Berlin conducted the initial validation and performance assessment of the measurement system. For this purpose, the animals were housed at the research site described in Section 2.1. During the preparatory research phase, three sensor prototypes and four halter designs were tested, as were ten sensor positions on the animals, in order to determine the optimal configuration of all components. This process enabled sufficient data to be collected for analysis and evaluation for the present study. The final system chosen was designed to automate the monitoring and classification of three key target sheep behaviours: rumination, resting/idling, and eating. The system consists of three main components: a data acquisition unit (sensor device); a data storage system; and a data processing module (algorithmic signal evaluation).

**Figure 1:**
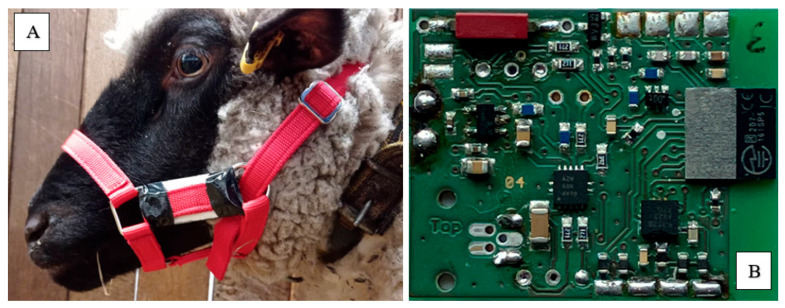
(**A**) The halter-integrated sensor positioned on the Masseter muscle below the zygomatic bone; (**B**) microcontroller with sensor unit.

The sensor device measured 70 × 35 × 11 mm (L × W × H) and weighed 40 g (see Figure 1A). It contained an accelerometer, a microcontroller, a standard lightweight lithium ion battery running at a voltage of 3.6 V, a project-specific (manufactured) circuit board, and a 3D printed plastic case for protection designed by BITSz Electronics. Data were recorded at a sampling frequency of 10 Hz, capturing acceleration values along the X, Y, and Z axes. Prior to collecting reference data, the electronic devices underwent testing for approximately four hours on different days and with different animals. The sensors were attached to the halter in the optimal position, above the masseter muscle and just below the zygomatic bone (see Figure 1A). The sheep were accustomed to wearing halters from the preparatory phase. Further technical specifications of the developed electronic device are provided below.

#### 2.2.2. Tri-Accelerometer Specifications

Sheep head movements were recorded using an integrated ADXL345 accelerometer from Analog Devices (Munich, Germany), which measures acceleration. This small, portable, non-invasive, 16-bit, three-axis sensor is suitable for measuring the intensity, duration, and frequency of particular animal behaviours. The sensor’s technical specifications include three axes (X, Y, and Z) and a serial peripheral interface (SPI). It provides high resolution with relatively low power consumption, operating at a voltage of 3.3 V. It offers a simple final schematic diagram with an addressing and data transfer protocol defined via special software developed by BITSz Electronics. From a technical standpoint, the algorithmic models were primarily optimised for the detection of rumination. The acceleration sensor was set to a sensitivity of ±2 g. Data were recorded at a sampling frequency of 10 Hz, capturing acceleration values along the X, Y, and Z axes.

#### 2.2.3. Microcontroller

The microcontroller employed in the system was the nRF52832 from Nordic Semiconductor (see Figure 1B). Its responsibilities included implementing system configuration (setting the acceleration sensor to a sensitivity of ±2 g), data acquisition, and wireless transmission. This 32-bit microcontroller is based on an ARM Cortex M4 processor architecture (which refers to the internal processing untit of the nRF52832 from Nordic Semiconductor) and combines program flash memory with buffers for intermediate storage. It also features flexible timers and counters with comparator modes, internal and external interrupts, a programmable serial port interface, a 12-bit analogue-to-digital converter, a programmable internal watchdog timer, and software-selectable power-saving modes. The device operates at a voltage of 3.3 V and uses a Bluetooth data transfer rate of 1 Mbit/s.

#### 2.2.4. System Software

The software comprising the two programs was the second main component after the hardware. Its implementation was based on the desired system functionalities. BITSz developed the entire software package. The electronic device was programmed to activate when the power button on the software installed on the laptop was pressed. This caused an LED on the sensor device to illuminate and begin recording accelerometer information based on X-, Y-, and Z-axis values.

The classification software developed by BITSz Eletronics in this project focuses solely on rumination, resting/idling time, and eating as target behaviours. This publication only considers the data obtained from the acceleration sensor. Following constructive programming, the software’s algorithms can distinguish between the three target behaviours by comparing the sensor’s signals with the “gold standard”—visual observations—of these behaviours.

#### 2.2.5. Data Storage and Power Supply for Electronic Components

The data were stored directly in the laptop’s permanent memory and transferred to the BITSz cloud server immediately after each measurement was taken.

The sensor device was powered by a separate lithium battery with a voltage of 3.6 V and a nominal capacity of 150 mAh. This enabled low power consumption of 2–4 µA during operation and less than 1 µA in standby mode. The receiver unit (converter/transducer), which had an antenna, was powered by being connected to a laptop via a USB cable (USB-A plug to micro-B USB plug).

#### 2.2.6. Halters and Sensor Deployment

As can be seen in Figure 1A, the sensor integrated into the halter was positioned below the zygomatic bone, on the masseter muscle. The device was attached to a polypropylene head halter for sheep (Albert Kerbl GmbH, Buchbach, Germany). Mounting the halter with the integrated sensor onto the sheep was not stressful or invasive.

#### 2.2.7. Software-Based Visual Behavioural Observation During Measurements

During each measurement period, two researchers conducted direct visual observations of the animals while the automated system was operating. These observations were used to validate and calibrate the sensor data. Behaviours were annotated using a time-stamped observation app developed by BITSz on a Lenovo YOGA Tablet 2-830F (Beijing, China) running Android 5.0.1. Using a fingertip, observers could select one of the following behaviours: ‘eating’, ‘ruminating’, ‘resting/idling’, ‘drinking’, ‘walking’, or ‘other (MISC)’. After entering a behaviour in the observation app, additional comments could be added. Behaviours including sniffing, scratching, and antagonistic interactions were classified within the ‘other/MISC’ category and supplemented with descriptive comments. The main behaviours evaluated in this paper were:

(A) Rumination behaviour, which includes the processes of regurgitating, re-chewing, re-salivating, and re-swallowing cud. This can be done while standing or lying down.
(B) Resting/idling behaviour, which includes the time an animal spends in a passive or resting state, without eating, ruminating, or interacting with other sheep. This behaviour can occur while standing or lying down, with the eyes open or shut, and with the head held upright or stretched towards the ground.
(C) Eating behaviour, which includes the time an animal spends consuming feed provided in or close to the trough.

The additional behaviours mentioned above were also considered during observations but are not included in this publication.

One observer evaluated one individual animal at a time, and the timestamps in the tablet software (BITSz Electronics, Zwickau, Germany) were synchronised with the sensor unit’s time to the second. Animal behaviour data were recorded in the observation software (BITSz Electronics, Zwickau, Germany) every second according to actual behaviour.

As the study comprised two parts—Part 1 focused on developing an accelerometer-based system and algorithm to automatically classify three key behaviours (rumination, resting/idling, and feeding), and Part 2 compared the duration of each behaviour under familiar and potentially stressful conditions —the materials and methods for each part are presented separately in the corresponding sections.

### 2.3. Specific Information Regarding Part 1: Testing and Validating 3D-Accelerometer-Based Classification of Sheep Behaviours

#### 2.3.1. Study Design

All sheep were kept under the conditions described in Section 2.1. This part of the study was conducted to evaluate the functionality and capacity of the entire system developed (including sensors, halters, signal transmission, algorithms, etc.) and all related parameters. The sheep wore the sensor device described in Section 2.2.6. for the duration of a four- to five-hour measurement session. The sensor device was worn consistently throughout these sessions.

#### 2.3.2. Data Collection and Analysis

Data collection took place between approximately 08:00 and 14:00, as it was expected that the animals would exhibit all the behaviours under study (eating, rumination, and resting/idling) during this period. Other behaviours were also observed in order to assess the quality of the sensor used in this study.

The prototype sensor device collected data at a sampling frequency of 10 Hz. The data was saved in CSV format on a laptop and subsequently processed in an Excel spreadsheet. To calculate the acceleration characteristic curve, the X, Y, and Z acceleration values determined by the acceleration sensor were used, taking the following parameters into account: signal magnitude area (SMA); magnitude of the signal vector (MSV); and the length and regularity of the ‘movement break’.

Both direct visual observations of individual sheep behaviour and observations made through camera recordings in Part 2 of the study (see Section 2.4.2) were viewed continuously at one-second intervals but evaluated at one-minute intervals. This procedure was chosen to avoid the observer having to record the animals’ behaviour every second, which could lead to errors in observation. It was therefore possible to determine the exact time that each animal spent performing each main activity, thereby minimising errors and achieving consistent results in model classification. These procedures resulted in 16,024 data points for Part 1 of this study. These data points were entered into classification models to analyse targeted behaviours, and segmented for each animal at one-minute intervals from the total datasets obtained.

#### 2.3.3. Digitalisation of the Visual Observation Protocol

Two researchers visually observed the animals directly at the research site during each four- to five-hour measurement using the aforementioned software (BITSz Electronics, Zwickau, Germany) installed on two tablets. The researchers stood outside the pen and looked through its bars. From this position, they were able to identify the behaviour of the sheep at any given moment without disturbing them. No prior training was required to perform these tasks, as individuals with experience of sheep could clearly distinguish the different behaviours. The observers were already familiar with using the BITSz observation app (Zwickau, Germany). The four- to five-hour timeframe was chosen because it allowed the researchers to observe nearly two full cycles of eating, followed by periods of rest and rumination.

#### 2.3.4. Development of Behavioural Classification Algorithms/Models

The classification model used to detect rumination behaviour was created using conventional algorithms but was also used to differentiate between resting/idling and eating behaviour without fine-tuning. These models were constructed using data from direct visual observations, which are considered the ‘gold standard’ for the purpose of this study. The displayed signal curves and amplitudes (X, Y, and Z lines) for each behaviour were taken into account. The models were then optimised for the classification of the three behaviours (rumination, resting/idling, and eating) using the full set of raw variables. The predictor variables were SMA, MSV, and movement breaks. The response variables were behaviours related to rumination, resting/idling, and eating. Behavioural classification was performed using a threshold-based approach applied to the acceleration data. First, cumulative movement activity was calculated from the envelope of the acceleration signal after removal of the gravitational component. The signal was represented as a resultant vector combining the x, y, and z axes to minimise the influence of sensor orientation. In parallel, higher-frequency components (“noise”) within the overall movement activity were isolated from the signal envelope using smoothing procedures with experimentally determined parameters. Rumination was then distinguished from other behaviours based on these two signal characteristics: periods of relatively high cumulative movement activity combined with relatively low high-frequency activity were classified as rumination, whereas eating and resting/idling exhibited different combinations of cumulative activity and signal variability.

#### 2.3.5. Model Validation (Sensitivity, Specificity, Accuracy, and Precision)

To investigate the reliability of the data provided by the sensor and assess its suitability for the automated detection of rumination, resting/idling, and eating, various performance parameters were calculated and evaluated alongside the visual observation data. This approach required the calculation of certain values, including the true positives (TP) marker (the number of samples where the observed behaviour was correctly identified and classified automatically), the true negatives (TN) marker (the number of samples where other behaviours were correctly identified and classified automatically), the false positives (FP) marker (the number of samples where other behaviours were incorrectly classified as the observed behaviour), and the false negatives (FN) marker (the number of samples where the observed behaviour was incorrectly classified as another behaviour). Determining these markers enhanced the performance of the classification models, which was evaluated using overall accuracy (Equation (1)), sensitivity (Equation (2)), specificity (Equation (3)), and precision (Equation (4)) as well as Cohen’s kappa index (Equation (5)) were calculated according to Ikurior et al. [17]:

Overall accuracy = [(TP + TN)/(TP + FN + FP + TN)](1)

Sensitivity = TP/(TP + FN)(2)

Specificity = TN/(TN + FP)(3)

Precision = TP/(TP + FP)(4)

Kappa = (Po − Pc)/(1 − Pc)(5)

Here, Po is the overall accuracy, and Pc is the proportion of units that agree by chance.

#### 2.3.6. Statistical Analysis for Part 1

The performance of the system was evaluated by identifying the most appropriate classification models for each behaviour, and by measuring the error minute by minute during each measurement period, comparing the visually observed data (the ‘gold standard’) with the data automatically classified by the developed system. The data were statistically analysed using “https://www.R-project.org/ (accessed on 16 June 2023)”. For this technical section of the publication (Part 1), data from 58 measurement processes involving all five sheep between 2021 and 2023 were evaluated.

To investigate the reliability of the data provided by the sensor for the automated detection of rumination, resting/idling, and eating in sheep, various quality parameters were calculated. Calculating the results for true positives (TP), true negatives (TN), false positives (FP), and false negatives (FN), as well as the Cohen’s kappa coefficient, enabled various key figures to be calculated, such as the sensitivity, specificity, accuracy, and precision of the developed system. Further investigations were conducted to clarify the potential influence of individual differences between sheep (e.g., jaw movement patterns or head shape) on the system’s ability to correctly recognise behaviour. These parameter values were examined for each animal individually.

### 2.4. Specific Information Regarding Part 2: Assessing Behavioural Changes in Sheep Exposed to a Potential Stressor

#### 2.4.1. Study Design: Description of the Conditions Sheep Were Subjected to

**Figure 2:**
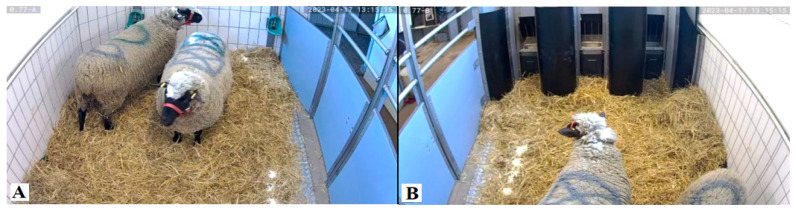
The view of the unfamiliar enclosure from camera A (image with letter (**A**) on the left) and camera B (image with letter (**B**) on the right).

**Figure 3:**
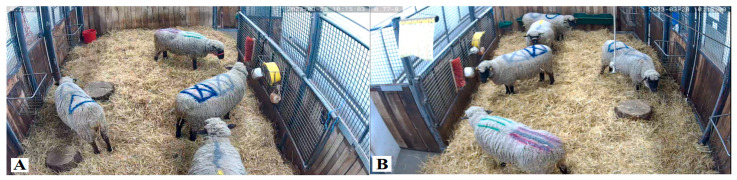
The view of the familiar enclosure from camera A (image with letter (**A**) on the left) and camera B (image with letter (**B**) on the right).

In Part 2 of the study, the duration of each of the three behaviours analysed was quantified for two separate housing conditions. The first condition (the ‘baseline condition’) involved keeping the sheep in the aforementioned familiar enclosure with their familiar group of five (see Figure 1A,B), while the second condition involved separating two of the sheep from their group and moving them to an unfamiliar enclosure. This condition will be referred to as the ‘unfamiliar condition’ from now on. The unfamiliar enclosure was located within the same animal facility and measured approximately 8.16 m^2^ (3.4 × 2.4 m), which complies with EU regulations for housing two sheep under laboratory conditions. The sheep were housed in the unfamiliar enclosure for a total of 72 h and then returned to their familiar group in their familiar enclosure. The unfamiliar enclosure was well lit and straw-bedded (Figure 2A,B). Animal husbandry practices, including feeding, were consistent in both conditions. Subjecting the sheep to the unfamiliar condition served the purpose of evaluating the effect of potential stress on behaviour. The 72 h measurement period was used to test the battery life capacity of the sensor unit. In the unfamiliar condition, pairs of sheep were placed in a room which housed other (unfamiliar) sheep in separate enclosures. The groups were housed together in the same room, but visual and tactile interaction was not possible. The other sheep were kept for an unrelated experimental study, but their presence was likely to have caused some stress. One week later, after the sheep had been returned to their familiar pen-mates and home enclosure, the separation procedure was repeated using the pair that had not previously been separated from the group. The pairs to be separated were chosen at random, not according to social rank or perceived compatibility. The sheep were marked with coloured and silver spray paint on their backs to facilitate identification during video observation (see Figure 3). This marking was renewed weekly to ensure it remained legible throughout the entire study period. In both cases, the sheep wore a halter with a sensor continuously throughout the experiment and were videotaped 24 h a day. Data for both the ‘baseline’ and ‘unfamiliar’ conditions were collected in March and April 2023. In the case of measurements taken in the unfamiliar enclosure, recordings commenced immediately after transitioning to the new enclosure to mark the onset of the stressor. Unlike Part 1, data for these trials were collected from four of the five sheep. One animal was excluded from the data collection but remained part of the group to ensure consistency. The fifth sheep was not included only because the set-up involved the relocation of sheep in pairs.

The experimental manipulation consisted of separating and relocating sheep in pairs, which the authors define as a “potential stressor”. Feeding regimes and general husbandry conditions were kept identical between the “baseline” and “unfamiliar” conditions. Accordingly, any behavioural differences observed between conditions were attributed to the change in location and/or temporary social separation, reduced enclosure size, and/or exposure to unfamiliar conspecifics housed in a separate enclosure within the same room. Many of these factors are known to elicit stress-related responses in sheep, as described in the introduction. An attempt was made to physiologically validate behavioural responses as indicators of stress by measuring heart rate variability. However, the use of a heart rate belt was discontinued due to frequent sensor displacement during postural changes (standing and lying), which resulted in unreliable signal acquisition. Alternative physiological measures were not employed, as these would have required additional disturbance of the animals (e.g., entering the enclosure to collect saliva or faecal samples). Consequently, physiological validation was not included in the final experimental design. The definition of the procedure as a “potential stressor” was therefore based on existing scientific literature indicating that alterations in rumination, resting/idling, and eating behaviour are commonly associated with stress-related responses in sheep. No definitive assessment of stress was made; rather, the animals were exposed to conditions considered capable of eliciting a potential stress response.

Data collection was conducted over a 24 h period, starting at either 10:00 or 11:00. This allowed the animals to exhibit all types of behaviour that occur daily under baseline or unfamiliar conditions.

In accordance with the method described in Section 2.3.2, 1440 data points were produced for Part 2 of the study. These data points were entered into classification models to analyse the targeted behaviours, and segmented for each animal at one-minute intervals from the total datasets obtained.

#### 2.4.2. Digitisation of Long-Term Observations Based on Video Recordings

The behaviour of the sheep was recorded for 24 h using two INSTAR IN-9408 2K+ PoE/LAN/PoE video cameras. These cameras were clamped onto the bars at opposite ends of the animal enclosure. The cameras have a night vision mode that activates automatically in low-light conditions. The video recordings were time-stamped for subsequent evaluation. A 256 GB MicroSDXC memory card was used to store the footage. Video footage was recorded continuously for 24 h over the course of four separate trials. This number of trials was conducted in order to study the behaviour of two animals per trial. This was necessary because the research team only had two sensors available. A total of 24 h of footage was analysed to allow multiple eating, rumination, and resting periods to be examined. Two observers assessed the sheep’s behaviour using the tablet’s software while continuously viewing 15 min video clips, carrying out the behavioural annotations of the videos. The time-stamped video recordings of the sheep were coded by playing each video at regular speed continuously.

For this part of the study, ‘rumination’ and ‘resting/idling’ behaviours were described as occurring in either the standing or lying down position.

If the animal’s behaviour was not clearly visible in the video recording (for example, if the sheep was facing the wall in a corner of the enclosure, obscuring its jaw and making it impossible for the observers to assess whether it was ruminating, resting, or idling), the observers entered ‘Other/MISC’ and commented that the animal’s head or behaviour was not clearly visible. In a few cases, technical malfunctions occurred during the 24 h measurement period, briefly interrupting the video recording. In such cases, the data continuously collected by the two sensor devices were used to fill the short gaps in the visual behavioural assessment data. However, the sheep’s behaviour was visible in most video recordings over the entire 24 h period (equivalent to 1440 min), despite occasional technical problems with one of the two video cameras. When one camera malfunctioned, it was usually possible to identify the sheep’s behaviour from the recordings of the other (functioning) camera. However, there were instances during the data collection process where an accurate determination of behaviour was not possible. The proportion of minutes in which the target animals were not visible remained below 5% (between 1% and 4.2%) in almost all cases, with only one exception. The lowest percentage of minutes for which reliance on the sensor was required was 1.0% for animal 1 under the ‘baseline’ condition. For animals 2, 4, and 5, the respective percentages under the baseline condition were 2.4%, 2.6%, and 3.4%, respectively. The highest percentage occurred under the ‘unfamiliar’ condition: 12.8% for animal 2, compared to 1.4%, 2.9%, and 4.2% for animals 1, 4, and 5, respectively.

#### 2.4.3. Statistical Analysis for Part 2

The data were analysed using R (www.R-project.org). The total number of minutes spent ruminating, resting/idling, and eating over a 24 h period was calculated for each of the four sheep, as well as the number of minutes spent standing or lying down ruminating or resting/idling. All data are presented as the mean ± standard deviation (SD), rounded to two decimal places. The duration of each behaviour was calculated for all four animals in their baseline state and under unfamiliar conditions. To test for differences between the two conditions, the data were first tested for normal distribution, and then the appropriate tests were applied (Student’s *t*-test for normally distributed data and the Wilcoxon rank sum test for non-normally distributed data). To test for differences between the two conditions, the data were first tested for normal distribution (Shapiro–Wilk normality test) and similar variances (F test to compare two variances), and then the appropriate tests were applied: paired Student’s *t*-test for normally distributed data and the paired Wilcoxon rank sum test for non-normally distributed data.

## 3. Results

### 3.1. Results of Part 1: Testing and Validating the Recognition of Sheep Behaviours Using a 3D Accelerometer

#### 3.1.1. Performance of the Discriminant Model Analysis

The statistical evaluation of the reliability of the system developed for the automated recognition of three key sheep behaviours (rumination, resting/idling, and eating) is presented in Table 1 for all five animals combined. Resting/idling exhibited the highest accuracy (0.90), specificity (0.95), and precision (0.88), while rumination showed the highest sensitivity (0.89). The sensitivity for eating was moderate (0.59), but the specificity was high (0.94). The agreement value (Cohen’s kappa) was similar for rumination and resting/idling, at 0.73 and 0.76, respectively, but lower for eating behaviour, at 0.57.

Across the entire dataset (16,024 min of observation), automatic detection was most accurate for rumination, correctly identifying 89% of events (Table 1, ‘Sensitivity’) and producing false negatives in only 11% of cases. The results of the investigation into the potential influence of individual differences between sheep can be found in Table 2, Table 3 and Table 4, which cover rumination, resting/idling, and eating behaviour, respectively. The data presented in this study are openly available in “Archiv_Sensors.zip”; see the Supplementary Materials. 

##### Rumination Behaviour

Table 2 summarises the performance values obtained for the automatic detection of rumination in individual animals. Cohen’s kappa values ranged from 0.51 to 0.83. The best overall performance was achieved with animal 2, with the highest accuracy (0.92), sensitivity (0.94), specificity (0.90), and precision (0.87), as well as the best agreement (κ = 0.83). Animal 1, on the other hand, showed the poorest performance (accuracy = 0.76, kappa = 0.51). Most parameters were consistent across the animals, with accuracy values ranging from 0.76 to 0.92, sensitivity values ranging from 0.75 to 0.94, and specificity values ranging from 0.76 to 0.90. However, only the precision values varied considerably between animals, ranging from 0.58 (animal 3) to 0.87. Animal 3 exhibited abnormal mandibular movement due to missing or malformed molars.

**Table 2:** Analytical values for automatic detection of “rumination” per animal, including markers, performance parameters, and Cohen’s kappa.

Animal	Duration (min)	Observed	Detected	TP	TN	FP	FN	Accuracy	Sensitivity	Specificity	Precision	Cohen’s Kappa
1	3126	1284	1405	969	1406	436	315	0.76	0.75	0.76	0.69	0.51
2	3785	1542	1663	1447	2027	216	95	0.92	0.94	0.90	0.87	0.83
3	2620	339	532	309	2058	223	30	0.90	0.91	0.90	0.58	0.65
4	3237	1445	1514	1321	1599	193	124	0.90	0.91	0.89	0.87	0.80
5	3256	1180	1412	1100	1764	312	80	0.88	0.93	0.85	0.78	0.75

Thus, if the data derived from the measurements conducted with animal 1 were theoretically excluded, the vast majority of the accuracy, sensitivity, and specificity values for rumination would be above 90%. These results demonstrate that performance parameters and sensor data quality for rumination vary only slightly depending on the animal.

##### Resting/Idling Behaviour

Table 3 shows the analytical results for the automatic detection of resting/idling behaviour in individual animals. Cohen’s kappa values ranged from 0.50 to 0.85. The strongest performance was observed with animal 5, with the highest accuracy (0.93), specificity (0.98), and precision (0.96), as well as the best agreement (κ = 0.85). By contrast, relatively low accuracy was observed with animal 4, which also had the lowest sensitivity (0.50) and precision (0.73), as well as the lowest Cohen’s kappa value (κ = 0.50). This indicates that the system had difficulty identifying resting/idling phases for that animal. Accuracy varied from 0.85 to 0.93. Sensitivity ranged from 0.50 to 0.90, while specificity was uniformly higher (0.88–0.98). Precision values ranged from 0.73 (animal 4) to 0.96 (animal 5), indicating that most detected resting/idling events were correctly classified.

**Table 3:** Analytical values for automatic detection of “resting/idling” per animal, including markers, performance parameters, and Cohen’s Kappa.

Animal	Duration (min)	Observed	Detected	TP	TN	FP	FN	Accuracy	Sensitivity	Specificity	Precision	Cohen’s Kappa
1	3126	958	1006	748	1910	258	210	0.85	0.78	0.88	0.74	0.65
2	3785	1148	1016	938	2559	78	210	0.92	0.82	0.97	0.92	0.81
3	2620	1530	1451	1378	1017	73	152	0.91	0.90	0.93	0.95	0.83
4	3237	718	486	357	2390	129	361	0.85	0.50	0.95	0.73	0.50
5	3256	1164	1029	987	2050	42	177	0.93	0.85	0.98	0.96	0.85

##### Eating Behaviour

Table 4 summarises the analytical performance of the automatic detection system in identifying eating behaviour in individual animals. Overall, the algorithm achieved accuracies ranging from 0.83 to 0.89 and Cohen’s kappa values ranging from 0.42 to 0.69. Regarding eating behaviour, the best overall performance could not be clearly attributed to a single animal but was distributed among several. Animal 2 exhibited the highest values for accuracy (0.89) and agreement (κ = 0.69), animal 4 exhibited the highest value for sensitivity (0.84), and animal 3 exhibited the highest values for specificity (0.97) and precision (0.86). The same trend was observed for the lowest values: animal 1 had the lowest values for sensitivity (0.39) and agreement (κ = 0.42), animal 4 had the lowest values for specificity (0.87) and precision (0.64), and animal 5 had the lowest value for accuracy (0.83).

**Table 4:** Analytical values for automatic detection of “eating” per animal, including markers, performance parameters, and Cohen’s kappa.

Animal	Duration (min)	Observed	Detected	TP	TN	FP	FN	Accuracy	Sensitivity	Specificity	Precision	Cohen’s Kappa
1	3126	644	349	249	2382	100	395	0.84	0.39	0.96	0.71	0.42
2	3785	853	855	647	2724	208	206	0.89	0.76	0.93	0.76	0.69
3	2620	639	380	328	1929	52	311	0.86	0.51	0.97	0.86	0.56
4	3237	704	927	593	2199	334	111	0.86	0.84	0.87	0.64	0.64
5	3256	749	429	318	2396	111	431	0.83	0.42	0.96	0.74	0.45

Notable variation in sensitivity was observed among the animals, ranging from 0.39 (animal 1) to 0.84 (animal 4), indicating variability in the system’s ability to correctly identify true eating events. However, specificity remained consistently high (0.87–0.97). Precision values ranged from 0.64 to 0.86.

**Figure 4:**
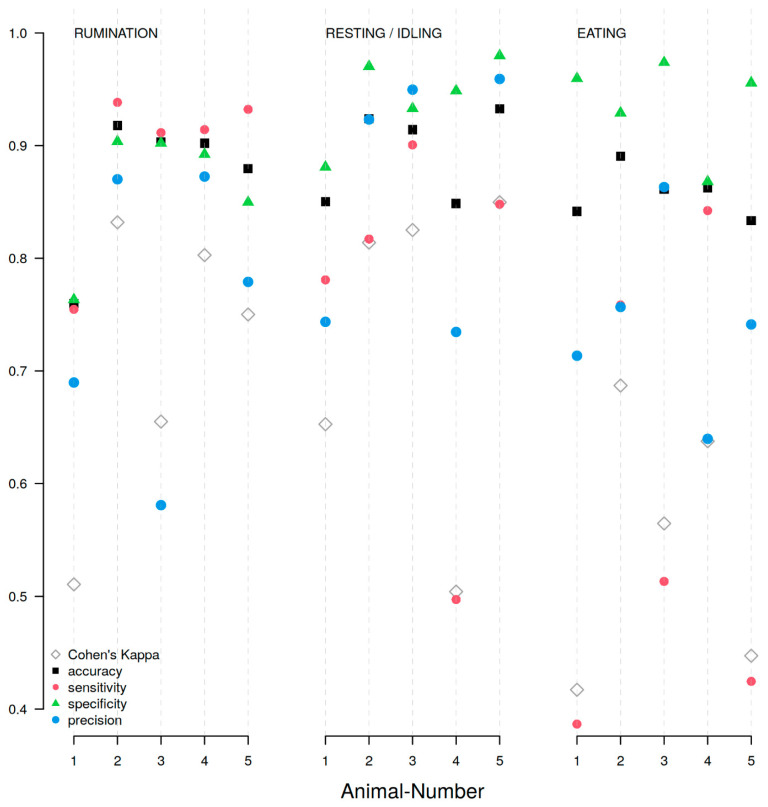
Quality parameters for behavioural detection of “rumination”, “resting/idling” and “eating” for each animal.

Figure 4 compares all of the values found in Table 2, Table 3 and Table 4 for each behaviour studied and for each individual animal. Overall, accuracy and specificity remained consistently high across all behaviours, while greater variability was observed in sensitivity and Cohen’s kappa values, particularly for eating. In general, rumination and resting/idling were more reliably detected and showed greater consistency between animals than feeding behaviour, for which sensitivity and kappa values were lower and more variable. However, all behaviours studied were reliably recorded in almost all animals. The accuracy, sensitivity, and specificity values for rumination and resting/idling behaviours were generally above 90%. Values for feeding behaviour detection were lower, ranging between 80% and 90%.

### 3.2. Results of Part 2: Assessing Behavioural Changes in Sheep Exposed to a Potential Stressor

#### 3.2.1. The Impact of Separating Sheep from Their Familiar Group and Relocating Them to an Unfamiliar Enclosure

Table 5 compares the mean durations of the observed behaviours (rumination, eating, and resting/idling) in baseline and unfamiliar conditions, regardless of whether the sheep was standing or lying down. A remarkable difference between the two conditions was observed in rumination duration. The mean rumination time decreased by 45.6% in the unfamiliar enclosure compared to the baseline condition, and this difference was significant (*p* = 0.0076). Conversely, the time spent resting/idling increased markedly by 47.9% when the sheep were exposed to the unfamiliar enclosure, and this difference was also significant (*p* = 0.0024). Furthermore, a non-significant decrease in eating duration (by 36.2%) was observed as a trend in the unfamiliar enclosure compared to the baseline condition.

#### 3.2.2. Impact of the Unfamiliar Condition on Behaviours in the Standing and Lying Position

Table 6 shows the differences in the duration of key behaviours between the baseline and unfamiliar conditions, distinguishing between standing and lying positions. Under unfamiliar conditions, the sheep spent significantly less time ruminating while lying down (*p* = 0.0036). Conversely, time spent resting/idling, in general, increased markedly (*p* = 0.0024), with a significant rise in idling time in the standing position (*p* = 0.0046).

## 4. Discussion

Part 1 of the study investigated the effectiveness of a novel 3D-accelerometer-based system using a nRF52832 microcontroller-powered accelerometer with Bluetooth-based wireless data transfer and an algorithm developed specifically for rumination detection in sheep. This system was capable of classifying three key behaviours with varying levels of reliability: rumination, resting/idling, and eating. Part 2 of the study found that changes in the duration of rumination and resting/idling behaviours are suitable parameters for automated alerts, as they were observed in response to the potential stressor. Rumination and resting/idling durations changed significantly when sheep were separated from their familiar group and moved to an unfamiliar environment in pairs, considered a “potential stressor”. These results suggest that such behavioural parameters could serve as the basis for automated alerts when deviations from individual (or perhaps group-level) baseline behaviours are detected. However, further validation with larger groups of sheep in different contexts is necessary to confirm their broader applicability.

The accelerometer-based classification system developed in this study achieved accuracy, specificity, and sensitivity values comparable to those reported in previous studies on automated monitoring of sheep behaviour. These previous studies are mentioned in the introduction. Numerous performance metrics indicate that the proposed system shows high reliability for the detection of certain behaviours, particularly rumination and resting/idling. Mean accuracies of 0.87 for rumination and 0.90 for resting/idling, together with substantial Cohen’s kappa values (κ = 0.73 and κ = 0.76, respectively), demonstrate that these behaviours can be identified consistently and with relatively high sensitivity and specificity across individual sheep. These findings support the suitability of the selected sensor position on the halter, across the cheek (masseter muscle), for capturing the characteristic jaw and head movements associated with these behaviours, both in standing and lying postures. In contrast, the system’s performance for eating behaviour was clearly lower, most notably reflected by a low sensitivity (0.39), despite a good overall accuracy (0.86). This discrepancy indicates that eating behaviour was frequently under-detected and highlights an important limitation of the current system. Accordingly, the results should be interpreted with caution—the overall system performance should not be considered uniformly high across all behavioural categories. Rather, the findings demonstrate insufficient performance for eating behaviour, indicating the need for further technical and algorithmic refinement. This need for refinement is supported by recent findings in goats, where Méndez et al. (2025) demonstrated that the choice of analytical model can substantially influence sensitivity values for feeding-related behaviours [22]. Several factors may explain the reduced sensitivity for eating. These may be related to biology, sensor technology, social dynamics, and methodological design. From a biological perspective, feeding behaviour in sheep involves subtle and complex movements of the head, jaw, and neck muscles. These movements are often minimally distinct from other behaviours such as rumination, grazing, or minor head movements, which may have produced comparatively weak and variable motion signals. As a result, the 3D accelerometer may have difficulty reliably distinguishing feeding from other similar activities, particularly under realistic, dynamic conditions where sheep move freely and briefly interrupt their feeding behaviour. From a technical standpoint, the algorithmic models were primarily optimised for the detection of rumination, which is characterised by highly regular and stereotyped jaw movements. Eating, by contrast, involves more variable patterns of head and jaw motion, depending on feed type, posture, and social context, making it more challenging to classify accurately using the current feature set. In this context, rumination—both in terms of its normal duration and potential reductions—may represent the most robust behavioural parameter for automated monitoring of health status and stress in sheep under controlled housing conditions, as it reflects both the quantity and quality of prior feed intake [23]. While the cheek-mounted sensor is optimally positioned for detecting stereotyped rumination movements, minor variations in head posture, body orientation, or wool coverage can weaken the signal during feeding. In addition, sheep often feed in close proximity, which can generate overlapping movement signals, which complicates behavioural classification. Similar challenges have been reported in previous studies [12,24], where sensor-based models achieved higher sensitivity for rumination than for feeding, and small variations in sensor placement or animal posture affected classification outcomes. From a methodological perspective, the layout of the feeding station in the experimental pen likely contributed to brief and irregular interruptions during feeding, particularly at the start of feeding bouts when sheep competed for access to the trough. These interruptions, often associated with agonistic interactions, were difficult for human observers to annotate in real time and may have introduced inconsistencies in the reference labels. Addressing this limitation is challenging, as the human observer needs to assess which behaviour the sheep is showing and will need time to find the appropriate key on the tablet. Methodological factors related to annotation and algorithm design further influence sensitivity. Accurate detection of feeding requires extensive and clearly labelled training data, yet such datasets are often limited due to the complexity and variability of feeding patterns. Conventional algorithms rely on manually defined features—such as head inclination, movement amplitude, and speed—which may capture general movement patterns but fail to represent subtle individual differences. Feeding behaviour varies with age, breed, and management system [25], meaning that rigid feature definitions and standard algorithmic may not apply across all individuals or contexts. These challenges highlight the importance of further technical refinement, including improved feature extraction, larger and more representative training datasets, and potentially multimodal sensing approaches to enhance discrimination between feeding and other behaviours. Within the classification algorithm, behavioural decisions were first derived from multiple shorter analysis windows substantially longer than 1 s, but shorter than 1 min, with window lengths determined experimentally. The final behavioural label assigned to each minute was then obtained by a majority-vote procedure across these shorter windows. This approach allowed short-term variability and transient movements to be accommodated while preserving robustness at the minute level. While aggregation to 1 min intervals may reduce sensitivity to brief behavioural events, this limitation is unlikely to have substantially affected the detection of the prolonged behavioural changes observed in response to the experimental manipulation. Instead, minute-level aggregation improved signal stability and interpretability for longitudinal welfare assessment, which is the intended application of the system. Furthermore, several potential sources of misclassification may have influenced overall classification performance. The first is inter-individual behavioural variability. Sheep differ in jaw movement amplitude, chewing rhythm, head carriage, and feeding style (even under identical housing and feeding conditions), which can reduce classification accuracy. Additionally, small differences in sensor orientation, attachment tightness, or displacement may have had an impact. Finally, single-modality sensing cannot capture all aspects of feeding behaviour (e.g., bite force or precise jaw kinematics). Consequently, certain feeding events may have remained indistinguishable from other head or jaw movements when relying solely on acceleration data. Multimodal sensing approaches—such as combining accelerometry with acoustic sensors and/or gyroscopes—may therefore improve classification accuracy and reduce ambiguity in future system iterations [26].

Performance values would have been higher if animal number 1 had been excluded from the analysis. This animal unexpectedly produced the lowest values for almost all rumination and eating quality parameters. What sets animal number 1 apart from the other sheep is that she weighed the most and ranked highest in the social hierarchy. Her rumination and eating behaviour did not visually differ from those of the other sheep. The results for the animal that did not ruminate in a typical physiological manner, on the other hand, were unexpectedly good. To the authors’ knowledge, previous research has largely focused on sheep with normal jaw physiology. In real-world settings, however, sheep may exhibit dental irregularities or other conditions that alter rumination behaviour despite intact rumen health, making the inclusion of this animal particularly informative. Further validation, involving animals with typical and atypical rumination patterns, is necessary in order to fully assess the system’s robustness and its applicability under practical field conditions. The current results could mean that the algorithm is highly sensitive to individual differences in behaviour. In practical applications, this sensitivity may indicate that individual calibration or adaptive modelling could further improve detection accuracy. This, however, should be investigated using a larger sample size of sheep.

The second part of this study demonstrated that exposure to a potential stressor—separating sheep from their familiar enclosure in pairs and relocating them to an unfamiliar one—resulted in measurable and statistically significant behavioural changes. Importantly, these changes occurred in the absence of any alterations to feeding regimes or standard husbandry practices, strongly suggesting that the observed behavioural shifts were attributable to stress. The reduction in rumination, in particular, supports the hypothesis that this behaviour is a highly sensitive ethological indicator of stress in sheep [18]. These findings are consistent with multiple previous studies identifying rumination as a key behavioural stress-marker, with the difference that other studies found rumination to be the most responsive behavioural parameter under conditions of compromised welfare [18]. The use of a larger cohort of sheep would have allowed for a more in-depth analysis of which behaviour is the most sensitive ethological parameter.

Exposure to a potential stressor resulted in the greatest reduction in lying rumination time and a corresponding increase in standing resting/idling behaviour. This is in agreement with previous studies linking these behaviours to the emotional state of sheep [27]. Rumination in the lying position is generally associated with emotional and physical comfort [23], while increased idling in the standing position, as observed here, may indicate stress in response to a novel environment while separated from the familiar herd [21]. The increase in standing idle time aligns with previous findings that moderate to severe stressors trigger a shift from comfort-related behaviours such as lying rumination to more alert postures [28,29]. Importantly, these behavioural shifts occurred under potentially stressful conditions, suggesting that the selected parameters are potentially responsive enough to capture subtle changes in the mental state of animals, which might be of welfare relevance. Once these findings are validated using physiological parameters that are known to be indicative of stress, these findings could be used to develop an objective, non-invasive, commercially available tool for detecting stress in sheep in agricultural and research contexts. Automatically detecting behavioural deviations based on 3D accelerometer classification would advance stress assessment, requiring low cost and labour. In livestock and experimental settings, a reliable recognition of subtle stress-related behavioural changes will enable timely intervention, preventing more severe welfare issues. This is promising for both farms and laboratory settings, where farmers and scientists are ethically and (in many countries) legally responsible for reducing avoidable stress and pain as much as possible. Furthermore, such systems could also improve reproducibility and transparency in animal research, where stress-induced behavioural variation can confound experimental outcomes.

Limitations: As a proof-of-concept study, this work was conducted using a small number of animals (N = 5 in Part 1 and N = 4 in Part 2), which substantially limits the generalisability and statistical power of the findings. The authors recognize this as a major limitation to the current results. Although the classification performance results and the observed behavioural changes provide preliminary evidence of the system’s potential, they should be interpreted cautiously, as the impact of inter-individual variability in sheep breed, sex, body size, and wool length cannot be fully characterised with the current sample size. Larger-scale studies across diverse breeds, ages, sexes, management systems, and environmental conditions are necessary in order to determine the robustness and broader applicability of the proposed approach. Another limitation is that visual behavioural observations were used as the reference method for validating the algorithm. While standard practice in behavioural research, human observation is subject to observer bias and occasional misclassification and therefore may not represent a perfect gold standard. This limitation may have influenced reported performance metrics and further underscores the need for multi-modal validation strategies in subsequent research. A limitation of the present study is that the same animals were used both (1) to develop and validate the behaviour-recognition algorithm and (2) to evaluate behavioural responses under baseline and the potentially stressful condition. This approach may reduce the independence of the results and limit the generalisability of the findings to other sheep or management contexts.

Behavioural changes observed in response to the “potential stressor” were not concurrently validated against physiological stress indicators (e.g., cortisol concentrations, heart rate variability, or other biomarkers). Validation, for example through heart rate variability, would have provided an independent measure to confirm that changes in rumination, resting/idling, and eating were indicative of stress. However, the observed reductions in rumination and increases in resting/idling following separation and relocation to an unfamiliar environment (a condition considered a “potential stressor”) were sufficient at the proof-of-concept stage to show that these ethological parameters serve as useful indicators for automated monitoring.

Future studies should evaluate the sensor system in both commercial livestock and controlled experimental settings, with baseline behavioural data collected over multiple consecutive days (e.g., at least three days) to account for day-to-day variability and circadian effects. Animals should then be exposed to mild, moderate, and more severe stressors commonly encountered in livestock and experimental environments, such as social regrouping, transport, handling procedures, or changes in housing and feeding regimes. This would allow the development of stress-response profiles and thresholds that are relevant under real-world management conditions.

From a practical implementation perspective, future work should focus on optimising sensor attachment, battery life, and data transmission stability to ensure suitability for long-term use (meaning multiple days) and on-animal deployment with minimal handling. Integration of the system into existing laboratory animal science systems could be achieved by linking behavioural outputs to score sheets or decision-support platforms, enabling automated alerts when deviations from individual or group-level baseline behaviours are detected. A similar approach could be conducted for livestock herd management systems. Such integration could facilitate early identification of stress-related welfare issues and support targeted management interventions, such as pain relief in the context of laboratory animal science. The present study focused on the automated classification of three core behaviours. Expanding the behavioural repertoire to include additional activities such as walking, drinking, and social interactions (both agonistic and socio-positive) would improve the depth of welfare assessment and provide a more comprehensive behavioural profile. Incorporating machine learning approaches capable of individualised baseline modelling is essential in order to enhance system sensitivity and adaptability across different sheep. Collectively, these refinements would advance the system from a proof-of-concept towards a precise and objective tool for continuous welfare monitoring in sheep.

## 5. Conclusions

This study demonstrates the strong potential of a 3D-accelerometer-based monitoring system that incorporates a novel sensor equipped with a nRF52832 microcontroller for Bluetooth-enabled wireless data transmission and a dedicated classification algorithm to reliably detect key sheep behaviours—particularly rumination and resting/idling—and, with further technical refinement, eating behaviour. In addition, all three behaviours were identified as ethological parameters that respond sensitively to a potential stressor—the separation of sheep from their familiar group and enclosure in pairs and their relocation to an unfamiliar environment. This indicates that these ethological parameters may be suitable for triggering automated alerts when deviations from baseline durations of behaviours occur. Although based on a small sample size, these findings suggest that sensor-based behavioural monitoring represents a non-invasive, objective, and ethically sound approach for assessing stress and supporting welfare management in sheep. While further technical improvements and validation in larger and more diverse populations under different management systems are required, this study constitutes an important step toward the refinement of technologies to assess animal welfare, contributing to both scientific understanding and ethical practice in agricultural and laboratory contexts.

## Figures and Tables

**Table 1:** Analytical values for the automatic detection ability of the sensor-based system for the detection of target behaviours in the entire dataset. These values include true positives (TP), true negatives (TN), false positives (FP), and false negatives (FN), as well as the performance parameters accuracy, sensitivity, specificity, and Cohen’s kappa.

Behaviour	Duration (min)	Observed	Detected	TP	TN	FP	FN	Accuracy	Sensitivity	Specificity	Precision	Cohen’s Kappa
Rumination	16,024	5790	6525	5146	8854	1380	644	0.874	0.889	0.865	0.789	0.73
Resting/idling	16,024	5518	4988	4408	9926	580	1110	0.895	0.799	0.945	0.884	0.76
Eating	16,024	3589	2940	2135	11,630	805	1454	0.859	0.595	0.935	0.726	0.57

**Table 5:** Comparison of the mean duration of key sheep behaviours in the baseline and unfamiliar conditions (min/24 h), without distinguishing between standing and lying positions ($\overset{-}{x}$ = mean; SD = standard deviation).

Animal/ Behaviour	1	2	3	4	$\overset{-}{x}$	SD	1	2	3	4	$\overset{-}{x}$	SD	Paired *t*-Test
	Baseline Condition (min/24 h)						Unfamiliar Condition (min/24 h)						*p* =
**Rumination**	451	476	474	541	485.5	38.7	318	274	188	276	264	54.6	0.00764 **
**Resting/idling**	632	594	692	630	637	40.6	913	934	1064	859	942.5	86.9	0.00237 **
**Eating**	331	299	240	200	267.5	58.7	162	162	160	198	170.5	18.4	0.125 ^†^
**Other**	26	71	34	69	49.5	24	47	70	28	107	63	34	0.292
**Σ**	1440						1440						11,520

* (*p* < 0.05), ** (*p* < 0.01), ^†^ Wilcoxon signed rank exact test.

**Table 6:** Comparison of the mean duration of individual sheep behaviours in the familiar and unfamiliar enclosures (min/24 h), with a distinction between standing and lying positions ($\overset{-}{x}$ = mean; SD = standard deviation).

Animal/ Behaviour	1	2	3	4	$\overset{-}{x}$	SD	1	2	3	4	$\overset{-}{x}$	SD	Paired *t*-Test
**Rumination**	**Baseline Condition (min/24 h)**						**Unfamiliar Condition (min/24 h)**						***p*** **=**
Lying down	373	461	444	455	433.3	40.8	208	268	163	257	224	48.3	0.00357 **
Standing up	78	15	30	86	52.2	35.0	110	6	25	19	40.0	47.3	0.5914
**Resting/Idling**	**Baseline Condition (min/24 h)**						**Unfamiliar Condition (min/24 h)**						***p* ** **=**
Lying down	518	544	403	490	488.8	61.3	548	689	557	374	542	129.2	0.461
Standing up	114	50	289	140	148.2	101.2	365	245	507	485	400.5	121	0.00464 **

* (*p* < 0.05), ** (*p* < 0.01).

## Data Availability

The original data presented in the study are openly available in “Archiv_Sensors.zip" at https://1drv.ms/u/c/af5e68a048f0605c/IQDiE-uGPIMZRpMum7kp5gkwAT_dnU-17DX_NvLi3PIlKrI?e=Slsejj (accessed on 8 February 2026), reference number “RefineMonSupplementaryMaterials2026”.

## Supplementary Materials

The data presented in this study are openly available in “Archiv_Sensors.zip” at “https://1drv.ms/u/c/af5e68a048f0605c/IQDiE-uGPIMZRpMum7kp5gkwAT_dnU-17DX_NvLi3PIlKrI?e=Slsejj (accessed on 8 February 2026)”, reference number “RefineMonSupplementaryMaterials2026”. If the link is inaccessible, please contact stephanie.schneidewind@outlook.de, and the files will be provided directly.
